# Nonlinear and sex-specific associations of maternal vitamin D in early- and mid-pregnancy with childhood growth trajectories from birth to 6 years of age: a prospective cohort study in China

**DOI:** 10.3389/fnut.2026.1781274

**Published:** 2026-04-24

**Authors:** Heng Zhang, Yimeng Su, Zeyu Zhang, Chenghao Li, Daozhen Chen

**Affiliations:** 1 Wuxi Maternity and Child Health Care Hospital, Wuxi School of Medicine, Jiangnan University, Wuxi, Jiangsu, China; 2 Wuxi Higher Health Vocational Technology School, Wuxi, Jiangsu, China

**Keywords:** cohort study, group-based trajectory modeling (GBTM), growth trajectory, nonlinear association, vitamin D

## Abstract

**Objectives:**

To examine the associations of maternal vitamin D (VitD) concentrations in early- and mid-pregnancy with offspring growth trajectories from birth to 6 years of age, as well as childhood overweight.

**Methods:**

This study was a prospective observational cohort study including 1,100 mother–child dyads from the Wuxi Birth Cohort. Offspring weight-, height-, and BMI-for-age Z-scores (WAZ, HAZ, and BAZ) were collected from birth to 6 years of age. Growth trajectories were identified using group-based trajectory modeling (GBTM). Restricted cubic spline (RCS) analyses explored the nonlinear associations. Multivariable logistic regression was used to assess associations of maternal VitD tertiles (T1-T3) with growth trajectory groups and overweight risk, with exploratory analyses stratified by child sex.

**Results:**

Lower early pregnancy VitD concentrations (T1 vs. T2) were associated with higher odds of increasing HAZ (aOR, 1.84; 95% CI, 1.16, 2.92) and BAZ (aOR, 1.63; 95% CI, 1.09, 2.43) trajectories. Lower mid-pregnancy VitD concentrations were associated with higher odds of increasing WAZ (aOR, 3.21; 95% CI, 1.41, 7.34) and HAZ (aOR, 1.94; 95% CI, 1.03, 3.66) trajectories. Higher early pregnancy VitD concentrations (T3 vs. T2) were associated with higher odds of increasing BAZ, particularly among boys (aOR, 2.11; 95% CI, 1.16, 3.82). Sex-stratified analyses suggested stronger associations for WAZ- and BAZ-related trajectories in boys and HAZ-related trajectories in girls. RCS analyses showed nonlinear associations, with the lowest odds of adverse trajectories observed at 27.6–72.6 nmol/L in early pregnancy and 28.8–76.2 nmol/L in mid-pregnancy. Overweight at age 6 was nearly ten times more common in children with increasing vs. stable BAZ trajectories.

**Conclusion:**

Maternal VitD levels in early- and mid-pregnancy showed nonlinear and sex-specific associations with offspring growth trajectories. Both low and high maternal VitD levels during these gestational periods were associated with higher odds of unstable growth trajectories.

## Introduction

Vitamin D (VitD) is a fat-soluble secosteroid primarily synthesized in the skin through ultraviolet-B (UVB) radiation–induced photoconversion. Beyond its classical role in calcium–phosphate homeostasis and skeletal mineralisation, VitD also regulates cellular proliferation, differentiation, and maturation (1, 2). VitD deficiency affects an estimated 48% of the global population (3), and pregnant women are disproportionately affected, with 54% having circulating 25-hydroxyvitamin D (25OHD) concentrations below 50 nmol/L (4). Because trans-placental transfer is the sole source of fetal VitD, maternal VitD status is a major determinant of neonatal VitD stores (5). Accumulating evidence links maternal VitD insufficiency to impaired fetal growth and adverse postnatal metabolic programming (6–8). Consequently, understanding VitD status during pregnancy and its associations with offspring growth and long-term health represents a major public health concern.

Most previous studies have focused on VitD exposure during a single gestational window or relied on cross-sectional or short-term designs, thereby limiting the assessment of longitudinal growth trajectories. In the Dutch Generation R cohort, inadequate second-trimester VitD was associated with smaller third-trimester head circumference, femur length, and estimated fetal weight (7). Several large prospective cohort studies have reported associations between VitD deficiency and preterm delivery, low birth weight, and shorter birth length (7–9), although some studies have yielded null findings (10–12). With longer follow-up, Chen et al. observed that lower maternal 25OHD concentrations during pregnancy increased the odds of infant overweight/obesity at 1 year of age (8). Beyond single time-point anthropometric outcomes, longitudinal growth trajectories capture dynamic patterns of weight and length gain and provide a more comprehensive assessment of early growth, which may better predict later adiposity and metabolic risk (13, 14). Evidence from the Shanghai Birth Cohort suggests that higher third-trimester and cord blood 25OHD concentrations are linked to a lower risk of persistently high weight-for-age trajectories from birth to 4 years of age (15). However, data linking repeated prenatal VitD measurements with longitudinal trajectories of standardized child growth from birth through early childhood, particularly regarding potential sex-specific differences in growth patterns, remain limited.

Using data from the Wuxi Birth Cohort, we examined the associations between maternal VitD concentrations in early- and mid-pregnancy and offspring weight, length/height, and body mass index (BMI) trajectories from birth to 6 years of age, with analyses further stratified by child sex.

## Methods

### Study participants

**Figure 1:**
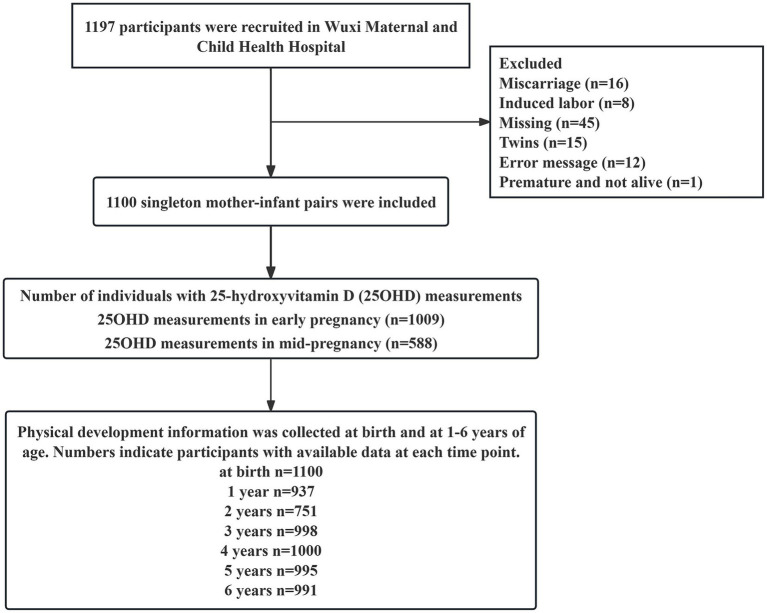
Flowchart of the study participant.

This study was based on a prospective observational cohort established at the Wuxi Maternity and Child Health Hospital in China, designed to investigate factors influencing maternal and infant health (16). Women were eligible if they (i) were residents of Wuxi City, (ii) intended to deliver at the study hospital between August 2017 and July 2019, (iii) delivered a singleton live birth, and (iv) were able to complete a structured questionnaire and provided written informed consent. Figure 1 summarizes the participant flow. Of the 1,197 enrolled women, 97 were excluded due to miscarriage (*n* = 16), termination of pregnancy (*n* = 8), twin pregnancy (*n* = 15), neonatal death (*n* = 1), loss to follow-up (*n* = 45), or critical data errors (*n* = 12), leaving 1,100 mother–infant pairs for analysis. Physical development data were collected from birth to 6 years of age. The number of participants with available anthropometric measurements at each time point is shown in Figure 1. Maternal serum 25-hydroxyvitamin D (25OHD) concentrations were measured on a voluntary basis.

The study protocol was approved by the Ethics Committee of Wuxi Maternity and Child Health Care Hospital (2022-01-0714-10) and registered with the Chinese Clinical Trial Registry (ChiCTR2300073310).

### 25-Hydroxyvitamin D measurements

Maternal serum samples were centrifuged within 30 min of collection, aliquoted, and stored at −80 °C. Total 25OHD concentration was determined by electrochemiluminescence immunoassay (ECLIA; Elecsys VitD total, Roche Diagnostics) following the manufacturer’s protocol (17, 18). The interassay coefficient of variation was < 5%.

### Anthropometric measurements

To capture changes in weight, height, and body mass index (BMI) in children across different ages, we selected weight-for-age z-score (WAZ), height-for-age z-score (HAZ), and BMI-for-age z-score (BAZ) as primary endpoints (19, 20). Weight and recumbent length or standing height were measured at birth and at 1, 2, 3, 4, 5, and 6 years of age by nurses who were blinded to maternal 25OHD status. WHO Anthro Plus (version 3.2.2) was used to calculate WAZ, HAZ, and BAZ according to the WHO growth standards (21).

### Covariates

Trained interviewers administered validated questionnaires during pregnancy and at follow-up visits. Maternal characteristics included age, height, pre-pregnancy weight, pre-pregnancy BMI, gestational weight gain (calculated as delivery weight minus pre-pregnancy weight), education, per-capita monthly household income, season of delivery, gravidity, parity, passive smoking during pregnancy, and use of VitD and folic acid supplements. Infant covariates at 1 year of age comprised VitD supplementation, daily outdoor activity time, and daily sleep duration. These baseline variables were selected *a priori* as potential confounders based on evidence from previous cohort studies and systematic reviews examining maternal VitD status and offspring growth outcomes, and were adjusted for in the multivariable analyses (17, 22).

### Statistical analyses

Continuous variables were presented as mean ± standard deviation (SD) and categorical variables as number (percentage). Group differences were assessed using the unpaired Student’s t-test for continuous variables and the chi-square test for categorical variables.

Group-based trajectory modeling (GBTM) identified distinct longitudinal trajectories of WAZ, HAZ and BAZ from birth to 6 years of age (23). Models with varying numbers of trajectory groups (1–5) and polynomial orders (linear, quadratic, cubic) were fitted. The optimal model was selected based on the following criteria: (1) the lowest Bayesian Information Criterion (BIC), indicating better model fit; (2) adequate trajectory group size (≥5% of the sample); (3) average posterior probability ≥0.70 for each group, indicating acceptable classification accuracy; and (4) clinical interpretability of the identified trajectories (24). Entropy was used to further assess classification quality, with values closer to 1 indicating better separation between trajectory groups. Participants were assigned to the trajectory group with the highest posterior probability. The trajectory closest to the null Z-score was used as the reference group. The optimal number of trajectory groups differed across growth indicators. For WAZ and HAZ, a three-group model provided the best balance between model fit, group size, classification quality, and interpretability. Although models with more groups yielded lower BIC values, they were not retained because at least one trajectory group comprised less than 5% of the sample. For BAZ, the two-group model met all model selection criteria. Models with three or more groups resulted in small subgroup sizes (<5%) and less stable or clinically interpretable trajectories. Therefore, a two-group model (stable vs. increasing) was selected. Details of model selection and classification statistics were presented in Supplementary Table 1.

Maternal VitD concentrations were categorized into cohort-specific tertiles (T1–T3) based on the 33rd and 66th percentiles, with T2 as the reference (Supplementary Table 2). Logistic regression was used to examine associations between growth trajectory groups from birth to 6 years of age and maternal VitD tertiles in early- and mid-pregnancy. Using restricted cubic splines (RCS) and a multivariate logistic regression model, the relationship between maternal VitD concentrations in early and mid-pregnancy and the risk of adverse growth trajectories was analyzed after adjusting for confounding variables. Exploratory analyses were additionally performed stratified by child sex to assess potential sex-specific patterns.

Overweight was defined as BAZ > +1 SD, corresponding to overweight risk for children aged 0–5 years and overweight for children aged 5–19 years, based on WHO standards (25–27). Logistic regression was used to examine associations between maternal VitD at different gestational time points and the risk of overweight from ages 1 to 6 years. At age 6 years, the proportions of overweight and non-overweight children within each BAZ trajectory group were calculated and depicted as stacked bar charts.

### Sensitivity analyses

Baseline characteristics of participants with and without 25OHD measurements were compared, and variables differing at *p* < 0.10 were included in fully adjusted models. Missing data at the follow-up of children at 2 years of age were assessed by comparing baseline characteristics between participants with and without available anthropometric data.

To assess the robustness of our findings, we conducted a sensitivity analysis including only those children with complete growth and adiposity measurements at all follow-up visits from birth to 6 years of age. The same multivariable logistic regression models, as in the primary analysis, were applied to the complete-case dataset.

The GBTM was performed using latent class mixed models (LCMM) with the hlme function in R. The analysis was conducted in R version 4.3.3 using the lcmm package for trajectory modeling and ggplot2 package for visualization. Since the same population was involved in multi-time-point detection, *p*-values were adjusted by Benjamini−Hochberg method to decrease FDR. All *p*-values were derived from two-tailed tests, and *p* < 0.05 was considered statistically significant.

## Results

### Participant characteristics

Participant characteristics were summarized in Table 1, with baseline characteristics of participants with and without VitD measurements shown in Supplementary Table 3. Most women were aged 25–34 years (87.45%), nulliparous (69.4%), had a normal pre-pregnancy BMI (67.82%), and held a college degree or higher (43.64%). Infant sex distribution was similar between males (47.5%) and females (52.5%). Prenatal supplementation was reported by 51.3% of women for VitD and 57.2% for folic acid. A total of 1,009 (91.7%) and 588 (53.5%) women were assessed for VitD in early and mid-pregnancy, respectively. The mean ± SD VitD concentration was 37.03 ± 14.27 nmol/L in early pregnancy and 43.10 ± 20.43 nmol/L in mid-pregnancy. Missing data at the 2-year child follow-up were evaluated. Most baseline characteristics were similar between participants with vs. without 2-year follow-up data; differences were observed only for pre-pregnancy BMI and season of delivery (Supplementary Table 4).

**Table 1:** Demographic characteristics of the pregnant women and infants (*n* = 1,100).

Characteristics		*n* (%)/(Mean ± S)
Maternal		
Maternal age (y)	< 25	82 (7.45)
	≥ 25, < 35	962 (87.45)
	≥ 35	56 (5.09)
Pre-pregnancy BMI (kg/m2)	Wasting	159 (14.45)
	Normal	746 (67.82)
	Overweight	157 (14.27)
	Obesity	38 (3.45)
Gestational weight gain	Excessive	235 (21.36)
	Suitable	457 (41.55)
	Too few	408 (37.09)
Educational level (y)	< 9	96 (8.73)
	≥ 9, < 16	524 (47.64)
	≥ 16	480 (43.64)
Per capita household income (CNY/month)	< 2,500	156 (14.18)
	≥ 2,500, < 5,000	775 (70.45)
	≥ 5,000	169 (15.36)
Gravidity	Primigravida	533 (48.45)
	Multigravida	567 (51.55)
Parity	Nulliparous	763 (69.36)
	Multiparous	337 (30.64)
Mode of delivery	Vaginal delivery	634 (57.64)
	Cesarean section	466 (42.36)
VitD supplementation	No	536 (48.73)
	Yes	564 (51.27)
Folic acid supplementation	No	471 (42.82)
	Yes	629 (57.18)
Passive smoking in gestation	No	910 (82.73)
	Yes	190 (17.27)
Child		
Gestational age (weeks)	—	38.88 ± 1.45
Birth weight (kg)	—	3.32 ± 0.45
Infant sex	Boy	522 (47.45)
	Girl	578 (52.55)
Season of delivery	Spring	318 (28.91)
	Summer	188 (17.09)
	Autumn	298 (27.09)
	Winter	296 (26.91)
VitD supplementation^a^	No	53 (4.80)
	Yes	865 (75.60)
Daily outdoor time^a^	< 1 h	89 (8.00)
	≥ 1 h	832 (75.60)
Daily sleep duration^a^	< 11 h	32 (2.90)
	≥ 11 h	758 (68.90)

a: Offspring at 1 year old.

### Long-term effects of maternal VitD levels in early and mid-pregnancy on growth trajectories from birth to 6 years of age

**Figure 2:**
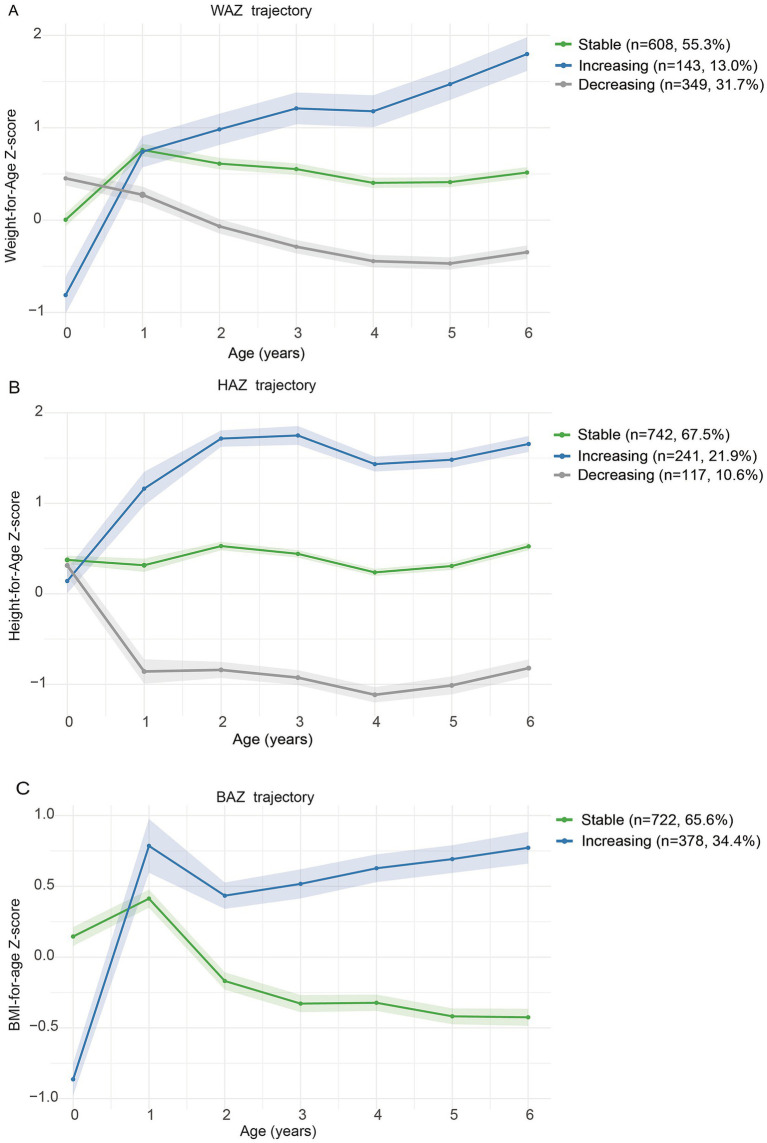
Growth trajectories of WAZ, HAZ, and BAZ from birth to 6 years of age. WAZ **(A)**, HAZ **(B)**, and BAZ **(C)** trajectories are shown by trajectory group. Shaded areas indicate 95% confidence intervals. Colors indicate trajectory patterns: stable (green), increasing (blue), and decreasing (gray, WAZ and HAZ only). Sample sizes and percentages are shown in the legend.

We first identified distinct longitudinal growth patterns using GBTM (Figure 2; Supplementary Figure 1). WAZ and HAZ were categorized into three groups: stable, increasing, and decreasing. BAZ was categorized into two groups: stable and increasing. Multivariable logistic regression models were then fitted to examine associations between maternal VitD status and offspring growth trajectories (Table 2). In the total sample, the lowest VitD tertile in early pregnancy was linked to increasing HAZ (aOR: 1.84, 95% CI: 1.16, 2.92) and BAZ (aOR: 1.63, 95% CI: 1.09, 2.43) trajectories. In mid-pregnancy, the lowest tertile was associated with increasing WAZ (aOR: 3.21, 95% CI: 1.41, 7.34) and HAZ (aOR: 1.94, 95% CI: 1.03, 3.66) trajectories. Maternal VitD in the highest tertile was generally not associated with WAZ or HAZ trajectories, with only early pregnancy BAZ showing slightly higher odds of following an increasing trajectory in the total sample (aOR: 1.55; 95% CI 1.04, 2.31) and in boys (aOR: 2.11; 95% CI 1.16, 3.82). Sensitivity analyses using complete-case data yielded similar results, with some attenuation of effect estimates (Supplementary Table 5).

**Table 2:** Adjusted odds ratios (95% CI) for offspring WAZ, HAZ, and BAZ trajectory groups at ages 1–6 years according to maternal vitamin D tertiles during early and mid-pregnancy, presented for the total sample and stratified by sex.

Trajectory groups	Early pregnancy			Mid-pregnancy		
	Adjusted OR (95% CI)			Adjusted OR (95% CI)		
	Total	Boys	Girls	Total	Boys	Girls
T 1 vs T 2						
WAZ						
Stable	Reference	Reference	Reference	Reference	Reference	Reference
Increasing	1.48 (0.84–2.62)	2.20 (1.00–4.80)*	1.38 (0.56–3.39)	3.21 (1.41–7.34)**	3.35 (1.12–10.06)*	2.60 (0.70–9.65)
Decreasing	0.87 (0.58–1.31)	1.32 (0.70–2.48)	0.61 (0.35–1.08)	1.17 (0.70–1.95)	1.28 (0.57–2.92)	1.07 (0.54–2.14)
HAZ						
Stable	Reference	Reference	Reference	Reference	Reference	Reference
Increasing	1.84 (1.16–2.92)**	1.48 (0.77–2.84)	2.36 (1.20–4.64)*	1.94 (1.03–3.66)*	0.96 (0.38–2.44)	3.19 (1.25–8.12)*
Decreasing	1.58 (0.88–2.84)	2.65 (0.99–7.07)	1.14 (0.52–2.50)	1.55 (0.72–3.32)	0.73 (0.17–3.13)	3.07 (1.14–8.27)*
BAZ						
Stable	Reference	Reference	Reference	Reference	Reference	Reference
Increasing	1.63 (1.09–2.43)*	1.78 (0.99–3.19)	1.56 (0.90–2.73)	1.29 (0.77–2.15)	1.09 (0.49–2.42)	1.60 (0.78–3.29)
T 3 vs T 2						
WAZ						
Stable	Reference	Reference	Reference	Reference	Reference	Reference
Increasing	1.00 (0.55–1.83)	1.19 (0.50–2.80)	1.11 (0.54–2.27)	1.46 (0.60–3.55)	0.93 (0.26–3.35)	2.42 (0.64–9.19)
Decreasing	0.78 (0.52–1.17)	1.21 (0.65–2.25)	0.72 (0.32–1.62)	0.78 (0.47–1.30)	0.57 (0.25–1.31)	1.05 (0.54–2.06)
HAZ						
Stable	Reference	Reference	Reference	Reference	Reference	Reference
Increasing	1.23 (0.77–1.99)	1.16 (0.59–2.29)	1.11 (0.54–2.27)	1.53 (0.80–2.93)	1.37 (0.53–3.51)	1.63 (0.62–4.32)
Decreasing	1.05 (0.57–1.92)	2.01 (0.72–5.58)	0.72 (0.32–1.62)	1.45 (0.67–3.13)	1.46 (0.41–5.22)	1.65 (0.60–4.53)
BAZ						
Stable	Reference	Reference	Reference	Reference	Reference	Reference
Increasing	1.55 (1.04–2.31)*	2.11 (1.16–3.82)*	1.15 (0.65–2.02)	1.03 (0.61–1.75)	0.90 (0.38–2.12)	1.38 (0.68–2.82)

Trajectory groups (stable, increasing, decreasing) were identified using group-based trajectory modeling based on repeated measurements from ages 1 to 6 years. “Stable” trajectory group was used as the reference category. T1, T2, and T3 represent the lowest, middle (reference), and highest tertiles of maternal serum vitamin D concentrations, respectively. WAZ, weight-for-age z score; HAZ, height-for-age z score; BAZ, BMI-for-age z score. All models were adjusted for maternal age, pre-pregnancy BMI, gestational weight gain, educational level, per capita household income, parity, delivery mode, gestational age, feeding patterns, infant sex, and child-related covariates at 1 year old, including VitD supplementation, daily outdoor time, and daily sleep duration. Analyses stratified by sex did not include sex as a covariate. P-FDR indicates *p* values adjusted for multiple comparisons using the false discovery rate (FDR) method. *p < 0.05, **p < 0.01.

Sex-stratified analyses revealed that these associations were more pronounced in boys for WAZ and BAZ trajectories and in girls for HAZ trajectories (Supplementary Figure 1). Among boys, the lowest tertile in early pregnancy was associated with increasing WAZ (aOR: 2.20; 95% CI: 1.00, 4.80), and in mid-pregnancy with increasing WAZ (aOR: 3.35, 95% CI: 1.12, 10.06). Among girls, the lowest tertile in early pregnancy was associated with increasing HAZ (aOR: 2.36; 95% CI: 1.20, 4.64), and in mid-pregnancy with both increasing (aOR: 3.19; 95% CI: 1.25, 8.12) and decreasing HAZ (aOR: 3.07; 95% CI: 1.14, 8.27).

**Figure 3:**
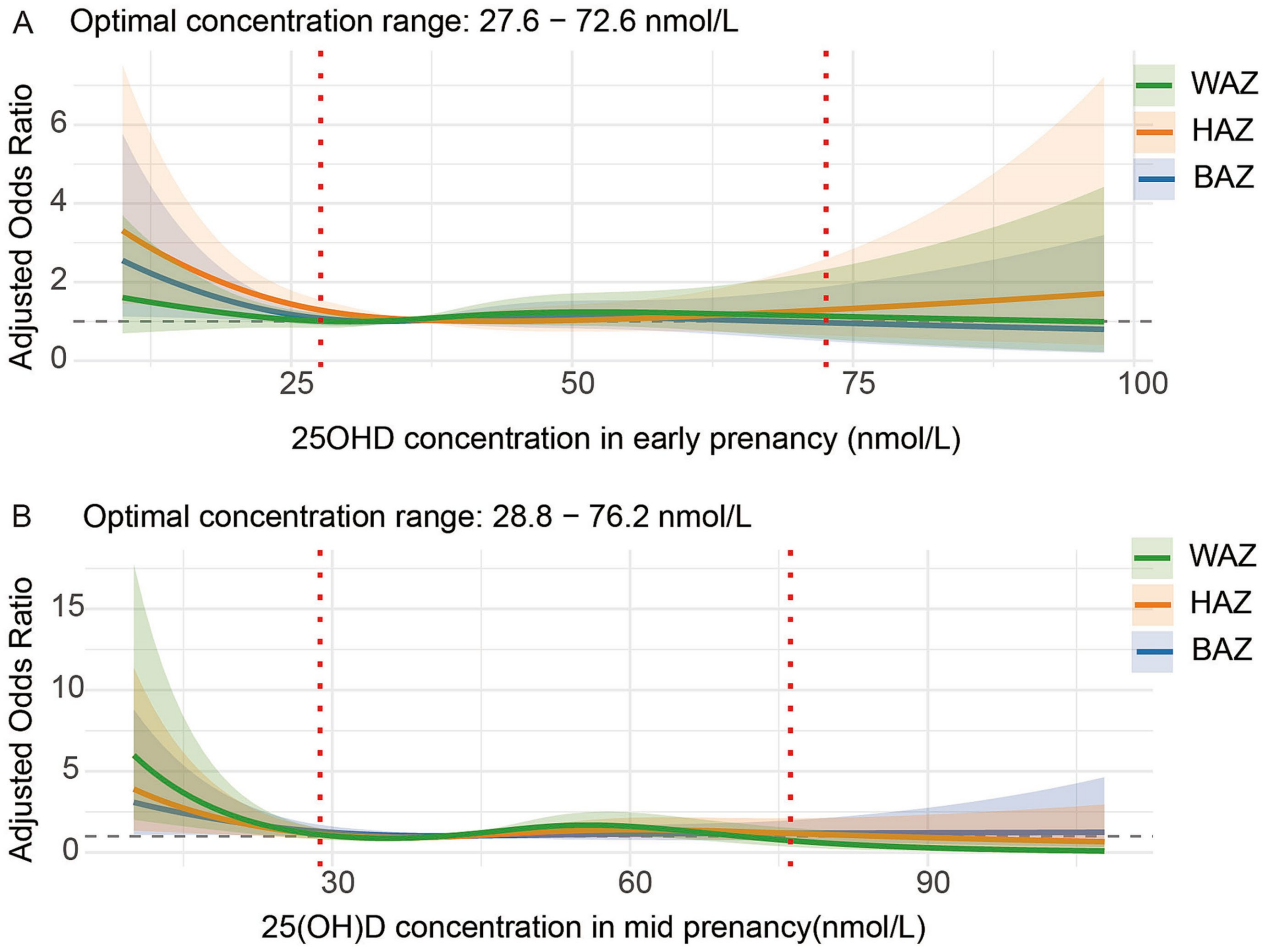
**(A)** Early pregnancy, **(B)** Mid-pregnancy. Dose–response relationship between maternal 25OHD concentrations during early and mid-pregnancy and adjusted odds of adverse child growth trajectories from birth to 6 years of age. Adjusted odds ratios (ORs, solid lines) and 95% confidence intervals (shaded areas) were derived from logistic regression models with restricted cubic splines. Maternal 25OHD concentrations are shown on the *x*-axis (nmol/L), and the *y*-axis shows the OR of adverse growth outcomes. Outcomes include WAZ (green), HAZ (orange), and BAZ (blue). Red dashed lines indicate the optimal 25OHD concentration ranges (early pregnancy: 27.6–72.6 nmol/L; mid-pregnancy: 28.8–76.2 nmol/L). ORs above 1 indicate increased risk of adverse growth. Models were adjusted for maternal age, pre-pregnancy BMI, gestational weight gain, educational level, household income, parity, delivery mode, gestational age, infant sex, infant feeding patterns, vitamin D supplementation, daily outdoor time, and sleep duration at age 1 year.

To assess dose–response patterns beyond tertile analyses, we used RCS models (Figure 3). In early pregnancy, maternal 25OHD concentrations of 27.6–72.6 nmol/L were associated with the lowest risk of adverse WAZ, HAZ, and BAZ trajectories in children (Figure 3A). In mid-pregnancy, the optimal 25OHD range was 28.8–76.2 nmol/L, which was similarly linked to minimal risk of adverse growth trajectories (Figure 3B).

### Associations of maternal VitD in early- and mid-pregnancy with offspring risk of overweight

**Figure 4:**
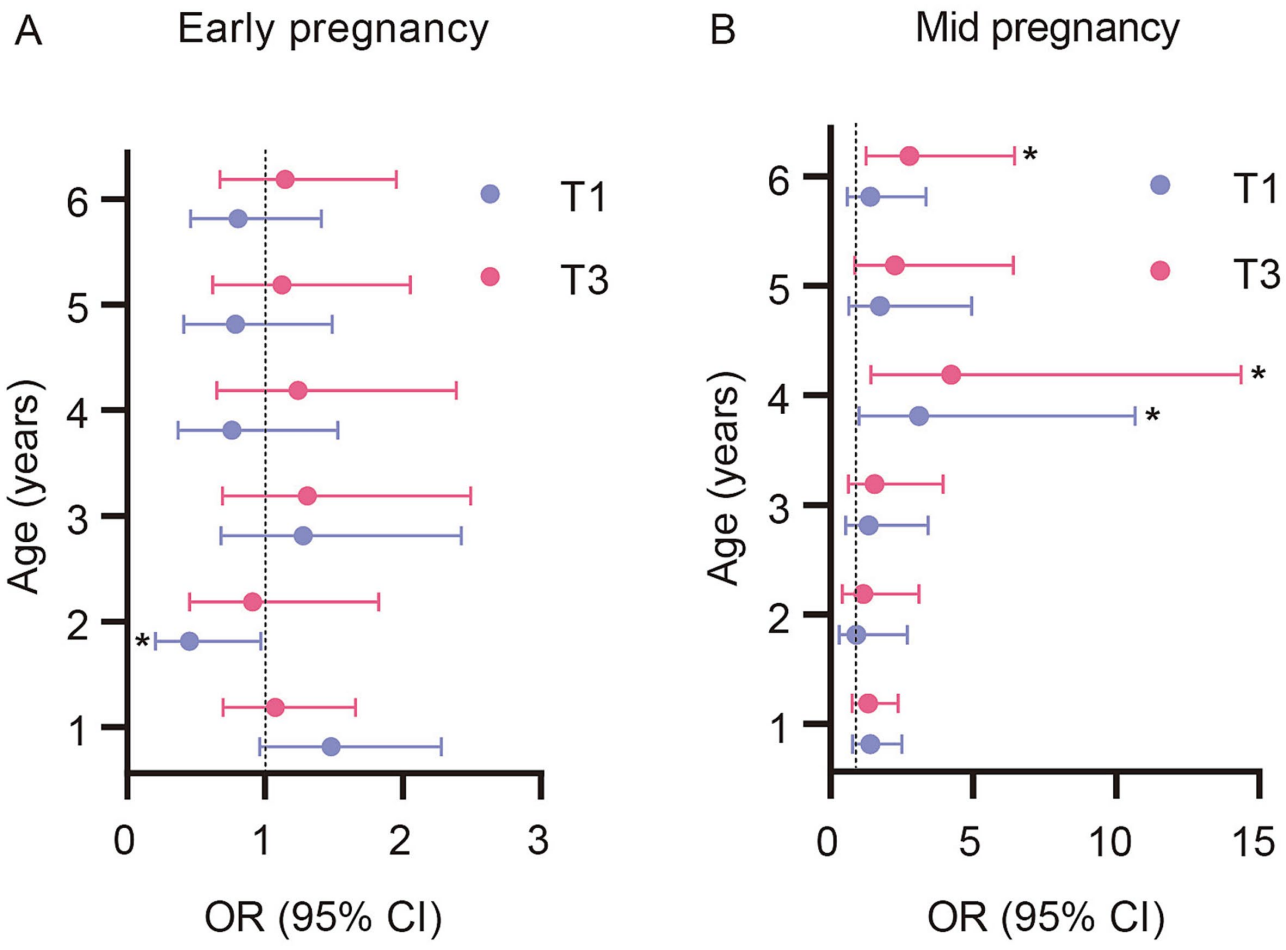
**(A)** Early pregnancy, **(B)** Mid-pregnancy. Adjusted odds ratios (95% CI) for elevated BMI-for-age z-score (BAZ > +1 SD) at ages 1–6 years according to tertiles of maternal vitamin D during early and mid-pregnancy. Odds ratios (ORs) and 95% confidence intervals (CIs) were estimated using multivariable logistic regression models, comparing the lowest (T1) and highest (T3) tertiles with the middle tertile (T2, reference). Models were adjusted for maternal age, pre-pregnancy BMI, gestational weight gain, education, household income, parity, delivery mode, gestational age, infant feeding patterns, and child-related factors at age 1 year (vitamin D supplementation, outdoor time, and sleep duration). *p < 0.05.

We next examined associations between the identified growth trajectories and overweight risk during follow-up (Figure 4; Supplementary Table 6). Higher mid-pregnancy VitD (T3 vs. T2) was associated with increased odds of offspring being at risk of overweight at age 4 (aOR: 4.22; 95% CI: 1.42, 14.37) and 6 years (aOR: 2.76; 95% CI: 1.25, 6.44). All associations were non-significant after FDR correction. At age 6 years, the prevalence of overweight was 34.6% among children in the increasing BAZ trajectory and 3.4% among those in the stable trajectory (34.6% vs. 3.4%) (Supplementary Figure 2; Supplementary Table 7).

Sex-stratified analyses were presented in Supplementary Table 8. Among boys, higher mid-pregnancy maternal VitD (T3 vs. T2) was associated with increased odds of being at risk of overweight at age 1 (aOR: 3.52; 95% CI: 1.28, 10.41; *p* = 0.018). Among girls, higher mid-pregnancy VitD was associated with increased odds at ages 4 (aOR: 10.69; 95% CI: 1.88, 111.58; *p* = 0.019), 5 (aOR: 16.67; 95% CI: 1.82, 351.61; *p* = 0.033), and 6 years (aOR: 14.72; 95% CI: 2.96, 114.51; *p* = 0.003). No consistent associations were observed for early pregnancy VitD in either sex. All associations were non-significant after FDR correction. Results were similar in complete-case analyses, although some associations were attenuated and no longer statistically significant (Supplementary Tables 9, 10).

## Discussion

This prospective observational cohort study revealed that maternal VitD status during early- and mid-pregnancy was associated with distinct offspring growth trajectories from birth to age 6 years of age. The associations varied by gestational timing and child sex. Notably, both lower and higher maternal VitD concentrations were linked to heightened odds of deviating from stable growth patterns.

Several maternal and child baseline characteristics captured in our cohort are known to influence offspring growth. Maternal pre-pregnancy BMI and gestational weight gain can affect fetal growth, subsequent childhood overweight and obesity risk, and may shape early postnatal weight trajectories (28, 29). Maternal VitD status during pregnancy has been linked to fetal growth patterns and birth outcomes such as size at birth (7), and VitD supplementation in pregnancy has been associated with improved neonatal anthropometry and early growth (30). Maternal age, education level, and household socioeconomic status have also been associated with child growth and long-term health outcomes, including nutritional status and healthcare access (31). Similarly, prenatal exposures such as VitD or folic acid supplementation and passive smoking may impact early growth outcomes and development (32). Child factors at birth, including gestational age, birth weight, and sex, are important determinants of subsequent growth trajectories. All of these variables were included as potential confounders in the multivariable models, allowing us to account for important sources of variation and focus on the independent associations between maternal VitD status in early- and mid-pregnancy and offspring growth trajectories.

Prior longitudinal studies examining prenatal VitD exposure and postnatal growth trajectories are limited and have reported heterogeneous findings (15, 33, 34). Differences in exposure windows, outcome definitions, and trajectory modeling approaches may partly explain these inconsistencies. Most previous studies focused primarily on BMI trajectories during infancy and early childhood and did not simultaneously evaluate multiple anthropometric dimensions (34, 35). By modeling WAZ, HAZ, and BAZ trajectories from birth to 6 years of age, our study provides a more comprehensive characterization of growth dynamics. Our findings suggest that maternal VitD status in early- and mid-pregnancy was associated with distinct growth patterns rather than uniformly influencing body size at a single age.

The observed associations between maternal VitD concentrations and offspring growth trajectories may reflect gestational timing-specific effects. In our study, lower mid-pregnancy VitD was associated with higher odds of increasing WAZ and HAZ trajectories, whereas higher early-pregnancy VitD was associated with increased odds of rising BAZ trajectories, particularly in boys. These bidirectional associations are consistent with the distinct physiological roles of VitD at different gestational stages. Early pregnancy is critical for placental formation and organ development; excessive VitD during this period may disrupt these processes and alter fetal programming of adiposity (7, 36). In contrast, mid-pregnancy involves rapid fetal skeletal growth and mineralisation, where VitD deficiency may impair linear growth and trigger compensatory postnatal weight gain (37). Clinically, these findings suggest that supplementation strategies should account for gestational timing and individual monitoring rather than applying a uniform approach across all trimesters.

The importance of early childhood growth trajectories is supported by substantial evidence linking rapid increases in BMI or weight during early life with an increased risk of later obesity (13, 14). Consistent with evidence linking lower prenatal VitD status to early-life overweight (8, 15), our findings showed that lower maternal VitD concentrations in early- and mid-pregnancy were associated with higher odds of offspring following an increasing trajectory. Children in the increasing BAZ trajectory had a higher prevalence of overweight at age 6 than those in the stable trajectory. While trajectory groups do not represent clinical diagnoses, they reflect differences in early growth patterns that are associated with later overweight risk (37, 38). In contrast to BAZ and WAZ, which primarily reflect weight gain and adiposity, HAZ is used to describe patterns of linear growth. Height-for-age z-scores relative to WHO growth standards are commonly used to assess linear growth and chronic deficits (e.g., stunting defined as HAZ < −2 SD), and do not inherently indicate adverse outcomes unless they reflect persistent faltering (39–41). Therefore, HAZ trajectories should be interpreted in the context of overall linear growth dynamics rather than directly linked to adiposity-related risks.

In addition, our analyses indicated that both insufficient and excessive maternal VitD in early- or mid-pregnancy were associated with an increased risk of unstable offspring growth trajectories. These findings challenge the simple linear dose–response hypothesis and may provide a novel perspective for reconciling inconsistent results reported in the literature. Our findings suggest that there may be an optimal concentration range for vitamin D’s effect on offspring. Although much of the literature has focused on the risks associated with maternal VitD deficiency, clinical evidence also suggests that excessive maternal VitD exposure may have negative perinatal consequences (42). A case report described severe neonatal hypercalcemia and prematurity following very high maternal VitD intake in late pregnancy, underscoring that both extremes of VitD status warrant attention rather than focusing solely on deficiency (42). Although deficiency is detrimental, exceeding an unstable threshold may also be unfavorable. Variations in gestational timing, outcome definitions, and analytical approaches may partly explain differences across studies.

Sex-specific effects were a central finding of our study. Boys were more sensitive to maternal VitD insufficiency, showing higher odds of increasing WAZ and BAZ trajectories, whereas HAZ trajectories in girls were more affected. Similarly, sex-stratified analyses of overweight risk suggested that higher mid-pregnancy 25(OH)D was linked to greater odds of overweight in girls at ages 4–6 and in boys at age 1, although these associations did not remain significant after FDR correction. The sex-specific growth patterns observed in our study align closely with findings from previous prospective cohort studies. Regarding weight- and BMI-related outcomes, evidence from the Spanish INMA cohort demonstrated that maternal VitD deficiency during pregnancy was associated with higher BMI z-scores, increased fat mass percentage, and elevated overweight risk in boys at 7 and 11 years of age, whereas no significant associations were observed in girls (43). Similarly, the Shanghai Birth Cohort reported that higher VitD levels in the third trimester and cord blood were associated with a reduced risk of high-increasing weight-for-age trajectories predominantly in boys, with effect estimates remaining null in girls (15). Collectively, these findings support a dimension-specific sexual dimorphism in the developmental programming effects of maternal vitamin D: weight- and adiposity-related growth appears more susceptible in male offspring.

Studies have suggested that male and female fetuses differ in placental gene expression and endocrine responses, leading to sexually dimorphic growth adaptations in utero (44). Epidemiological studies on developmental programming have further suggested that male fetuses may be more vulnerable to adverse intrauterine environments, while female fetuses may exhibit different adaptive growth responses (45). Given that VitD plays a role in placental function and skeletal development (46), differential sensitivity to maternal 25(OH)D by fetal sex is conceivable. Nevertheless, our sex-stratified analyses were exploratory and warrant confirmation in larger, multicentre cohorts as well as mechanistic studies to elucidate the underlying biological pathways.

Early childhood growth trajectories are increasingly recognized as predictors of later obesity and metabolic risk (13, 14). Our findings indicate that maternal VitD status in early- and mid-pregnancy may shape these trajectories, highlighting pregnancy as a sensitive window for nutritional exposures influencing postnatal growth. Using repeated anthropometric measurements from birth to 6 years of age and group-based trajectory modeling, we were able to capture dynamic, multidimensional growth patterns rather than relying on single time-point assessments. Current guidelines in China and many other countries generally recommend standard-dose VitD supplementation during pregnancy, with additional supplementation reserved for women with documented deficiency (47–49). While population-level strategies such as universal supplementation or food fortification have been implemented in some countries, including the United Kingdom and Nordic countries such as Sweden (50, 51), most existing guidelines do not account for potential trimester-specific effects or interindividual variability in VitD metabolism. Our results therefore underscore the need for further research to define maternal VitD ranges associated with optimal early childhood growth, and to evaluate whether gestational timing should inform supplementation strategies. Collectively, these findings contribute to refining prenatal nutrition guidelines and lay a foundation for population-based, individualized precision management by establishing optimal maternal VitD reference intervals specific to early- and mid-pregnancy.

This study has several limitations. First, as a prospective observational cohort, residual confounding cannot be excluded, and causal inferences cannot be drawn. Unmeasured factors, including childhood illness, dietary intake, sunlight exposure, and other environmental or behavioral influences, may have influenced the observed associations. Second, loss to follow-up and varying sample sizes across time points may have introduced selection bias. Missing data could reduce statistical power and affect the robustness of estimated trajectories. Third, maternal VitD was measured only in early and mid-pregnancy, limiting assessment of gestational timing effects. Finally, the study was conducted in a single region, which may limit generalisability, and validation in more diverse populations is warranted.

## Conclusion

Maternal VitD levels in early- and mid-pregnancy have nonlinear and sex-specific effects on offspring physical development. Both low and excessively high VitD concentrations in early- and mid-pregnancy were associated with an increased risk of unstable growth trajectories. These findings highlight potential gestation-stage-specific associations between maternal VitD and childhood growth patterns, suggesting that tailored monitoring of maternal VitD status during pregnancy may help inform future recommendations for maternal nutrition and support healthy child growth trajectories.

## Data Availability

The raw data supporting the conclusions of this article will be made available by the authors, without undue reservation.

## References

[1] Nakamichi Y Udagawa N Suda T Takahashi N. Mechanisms involved in bone resorption regulated by vitamin D. J Steroid Biochem Mol Biol. (2018) 177:70–6. doi: 10.1016/j.jsbmb.2017.11.005, 29146302

[2] Meenakshi Umar KSSAIC. Role of vitamin D beyond the skeletal function: a review of the molecular and clinical studies. Int J Mol Sci. (2018) 19:1618. doi: 10.3390/ijms19051618 PMC6032242 29849001

[3] Palacios C Gonzalez L. Is vitamin D deficiency a major global public health problem? J Steroid Biochem Mol Biol. (2014) 144:138–45. doi: 10.1016/j.jsbmb.2013.11.003, 24239505 PMC4018438

[4] Saraf R Morton SM Camargo CA Jr Grant CC. Global summary of maternal and newborn vitamin D status - a systematic review. Matern Child Nutr. (2016) 12:647–68. doi: 10.1111/mcn.12210, 26373311 PMC6860156

[5] Karras SN Wagner CL Castracane VD. Understanding vitamin D metabolism in pregnancy: from physiology to pathophysiology and clinical outcomes. Metabolism. (2018) 86:112–23. doi: 10.1016/j.metabol.2017.10.001, 29066285

[6] Santamaria C Bi WG Leduc L Tabatabaei N Jantchou P Luo ZC . Prenatal vitamin D status and offspring's growth, adiposity and metabolic health: a systematic review and meta-analysis. Br J Nutr. (2018) 119:310–9. doi: 10.1017/S0007114517003646, 29321080

[7] Miliku K Vinkhuyzen A Blanken LM McGrath JJ Eyles DW Burne TH . Maternal vitamin D concentrations during pregnancy, fetal growth patterns, and risks of adverse birth outcomes. Am J Clin Nutr. (2016) 103:1514–22. doi: 10.3945/ajcn.115.123752, 27099250 PMC5410992

[8] Chen C Zhou C Liu S Jiao X Wang X Zhang Y . Association between suboptimal 25-Hydroxyvitamin D status and overweight/obesity in infants: a prospective cohort study in China. Nutrients. (2022) 14:4897. doi: 10.3390/nu14224897, 36432582 PMC9698418

[9] Beck C Blue NR Silver RM Na M Grobman WA Steller J . Maternal vitamin D status, fetal growth patterns, and adverse pregnancy outcomes in a multisite prospective pregnancy cohort. Am J Clin Nutr. (2025) 121:376–84. doi: 10.1016/j.ajcnut.2024.11.018, 39577494 PMC11863332

[10] Ong YL Quah PL Tint MT Aris IM Chen LW van Dam RM . The association of maternal vitamin D status with infant birth outcomes, postnatal growth and adiposity in the first 2 years of life in a multi-ethnic Asian population: the growing up in Singapore towards healthy outcomes (GUSTO) cohort study. Br J Nutr. (2016) 116:621–31. doi: 10.1017/S0007114516000623, 27339329 PMC4967353

[11] Kokkinari A Dagla M Antoniou E Lykeridou A Iatrakis G. Are maternal vitamin D (25(OH)D) levels a predisposing risk factor for neonatal growth? A Cross-Sectional Study. Clin Pract. (2024) 14:265–79. doi: 10.3390/clinpract14010021, 38391407 PMC10887765

[12] Monier I Baptiste A Tsatsaris V Senat MV Jani J Jouannic JM . First trimester maternal vitamin D status and risks of preterm birth and small-for-gestational age. Nutrients. (2019) 11:3042. doi: 10.3390/nu11123042, 31847068 PMC6950733

[13] Giles LC Whitrow MJ Davies MJ Davies CE Rumbold AR Moore VM. Growth trajectories in early childhood, their relationship with antenatal and postnatal factors, and development of obesity by age 9 years: results from an Australian birth cohort study. Int J Obes. (2015) 39:1049–56. doi: 10.1038/ijo.2015.42, 26008137

[14] Liu JX Liu JH Frongillo EA Boghossian NS Cai B Hazlett LJ. Body mass index trajectories during infancy and pediatric obesity at 6 years. Ann Epidemiol. (2017) 27:708–15.e1. doi: 10.1016/j.annepidem.2017.10.008 29173577

[15] Chen C Zhou C Zhang J Tian Y Wang X Jiao X . Associations between longitudinal maternal and cord blood vitamin D status and child growth trajectories up to 4 years of age. Nutrients. (2024) 16:2410. doi: 10.3390/nu16152410, 39125291 PMC11313987

[16] Cai P He H Song X Qiu T Chen D Zhang H. Association between gestational arsenic exposure and infant physical development: a prospective cohort study. BMC Public Health. (2024) 24:2292. doi: 10.1186/s12889-024-19818-7, 39174974 PMC11342644

[17] Tous M Villalobos M Iglesias-Vazquez L Fernandez-Barres S Arija V. Vitamin D status during pregnancy and offspring outcomes: a systematic review and meta-analysis of observational studies. Eur J Clin Nutr. (2020) 74:36–53. doi: 10.1038/s41430-018-0373-x, 30683894

[18] Fang AP Long JA Zhang YJ Liu ZY Li QJ Zhang DM . Serum bioavailable, rather than Total, 25-hydroxyvitamin D levels are associated with hepatocellular carcinoma survival. Hepatology. (2020) 72:169–82. doi: 10.1002/hep.31013, 31677282 PMC7496975

[19] Arpe M-LH Rørvig S Kok K Mølgaard C Frandsen TL. The association between glucocorticoid therapy and BMI z-score changes in children with acute lymphoblastic leukemia. Support Care Cancer. (2015) 23:3573–80. doi: 10.1007/s00520-015-2718-5, 25894880

[20] Zhu Y Li J Wang L Qi Q Li S Cheng Y . Maternal gestational weight status and offspring physical growth status at birth, mid-childhood and early adolescence. Matern Child Nutr. (2025) 21:e70015. doi: 10.1111/mcn.70015, 40079394 PMC12150136

[21] Organization WH. Child growth standards: length/height-for-age, weight-for-age, weight-for-length, weight-for-height and body mass index-for-age: methods and development. (2006) Available online at: https://www.who.int/toolkits/child-growth-standards/standards/body-mass-index-for-age-bmi-for-age (Accessed December 4, 2025).

[22] Bi WG Nuyt AM Weiler H Leduc L Santamaria C Wei SQ. Association between vitamin D supplementation during pregnancy and offspring growth, morbidity, and mortality. JAMA Pediatr. (2018) 172:635–645. doi: 10.1001/jamapediatrics.2018.0302, 29813153 PMC6137512

[23] Nagin DS Jones BL Passos VL Tremblay RE. Group-based multi-trajectory modeling. Stat Methods Med Res. (2018) 27:2015–23. doi: 10.1177/0962280216673085, 29846144

[24] Liu W Luo D Zhou A Li H Covaci A Xu S . Prenatal exposure to organophosphate esters and growth trajectory in early childhood. Sci Total Environ. (2024) 912:169080. doi: 10.1016/j.scitotenv.2023.169080, 38052391

[25] Department of Nutrition WHO, Geneva, Switzerland, and Members of the WHO Multicentre Growth Reference Study Group WHO Child Growth Standards Based on Length/Height, Weight and Age. Oslo, Norway: Acta paediatrica (2006) doi: 10.1111/j.1651-2227.2006.tb02378.x 16817681

[26] Organization WH. Which cut-offs should we use for defining obesity risk in children? (2025) Available online at: https://www.who.int/news-room/questions-and-answers/item/child-growth-standards (Accessed December 5, 2025).

[27] Organization WH. Obesity and overweight (2025). Available online at: https://www.who.int/news-room/fact-sheets/detail/obesity-and-overweight? (Accessed December 5, 2025).

[28] Ma RCW Voerman E Santos S Patro Golab B Amiano P Ballester F . Maternal body mass index, gestational weight gain, and the risk of overweight and obesity across childhood: an individual participant data meta-analysis. PLoS Med. (2019) 16:e1002744. doi: 10.1371/journal.pmed.1002744 PMC6370184 30742624

[29] Oken E Kleinman KP Belfort MB Hammitt JK Gillman MW. Associations of gestational weight gain with short- and longer-term maternal and child health outcomes. Am J Epidemiol. (2009) 170:173–80. doi: 10.1093/aje/kwp101, 19439579 PMC2727269

[30] Amberntsson A Papadopoulou E Winkvist A Lissner L Meltzer HM Brantsaeter AL . Maternal vitamin D intake and BMI during pregnancy in relation to child’s growth and weight status from birth to 8 years: a large national cohort study. BMJ Open. (2021) 11:e048980. doi: 10.1136/bmjopen-2021-048980, 34598984 PMC8488702

[31] Hill B Parker HW Tovar A McCurdy K Vadiveloo M. Associations between pre-pregnancy BMI, gestational weight gain, and prenatal diet quality in a national sample. PLoS One. (2019) 14:e0224034. doi: 10.1371/journal.pone.0224034 PMC6799919 31626677

[32] Leonardi-Bee J Smyth A Britton J Coleman T. Environmental tobacco smoke and fetal health: systematic review and meta-analysis. Arch Dis Child Fetal Neonatal Ed. (2008) 93:F351–61. doi: 10.1136/adc.2007.133553, 18218658

[33] Jiang X Lu J Zhang Y Teng H Pei J Zhang C . Association between maternal vitamin D status with pregnancy outcomes and offspring growth in a population of Wuxi, China. Asia Pac J Clin Nutr. (2021) 30:464–76. doi: 10.6133/apjcn.202109_30(3).0013 34587706

[34] Amberntsson A Barebring L Winkvist A Lissner L Meltzer HM Brantsaeter AL . Maternal vitamin D status in relation to infant BMI growth trajectories up to 2 years of age in two prospective pregnancy cohorts. Obes Sci Pract. (2022) 8:10.1002/osp4.602:670–81. doi: 10.1002/osp4.602 36238227 PMC9535664

[35] Garden FL Marks GB Simpson JM Webb KL. Body mass index (BMI) trajectories from birth to 11.5 years: relation to early life food intake. Nutrients. (2012) 4:1382–98. doi: 10.3390/nu4101382, 23201761 PMC3497001

[36] Zhao R Zhou L Wang S Yin H Yang X Hao L. Effect of maternal vitamin D status on risk of adverse birth outcomes: a systematic review and dose–response meta-analysis of observational studies. Eur J Nutr. (2022) 61:2881–907. doi: 10.1007/s00394-022-02866-3, 35316377

[37] Belenchia AM Jones KL Will M Beversdorf DQ Vieira-Potter V Rosenfeld CS . Maternal vitamin D deficiency during pregnancy affects expression of adipogenic-regulating genes peroxisome proliferator-activated receptor gamma (PPARgamma) and vitamin D receptor (VDR) in lean male mice offspring. Eur J Nutr. (2018) 57:723–30. doi: 10.1007/s00394-016-1359-x 28004271 PMC6643277

[38] Daraki V Roumeliotaki T Chalkiadaki G Katrinaki M Karachaliou M Leventakou V . Low maternal vitamin D status in pregnancy increases the risk of childhood obesity. Pediatr Obes. (2018) 13:467–75. doi: 10.1111/ijpo.12267, 29377526

[39] Leroy JL Ruel M Habicht J-P Frongillo EA. Using height-for-age differences (HAD) instead of height-for-age z-scores (HAZ) for the meaningful measurement of population-level catch-up in linear growth in children less than 5 years of age. BMC Pediatr. (2015) 15:145. doi: 10.1186/s12887-015-0458-9, 26444012 PMC4595313

[40] Amaha ND. Determinants of height-for-age Z-score (HAZ) among Ethiopian children aged 0–59 months: a multilevel mixed-effects analysis. BMC Public Health. (2025) 25:1614. doi: 10.1186/s12889-025-22831-z, 40312674 PMC12044844

[41] Lundeen EA Stein AD Adair LS Behrman JR Bhargava SK Dearden KA . Height-for-age z scores increase despite increasing height deficits among children in 5 developing countries. Am J Clin Nutr. (2014) 100:821–5. doi: 10.3945/ajcn.114.084368, 25008854 PMC4135493

[42] Karacan Küçükali G Keskin M Savaş Erdeve Ş Çetinkaya S. Perinatal outcomes of high-dose vitamin D administration in the last trimester. J Turkish Soc Obstetric Gynecol. (2021) 18:159–62. doi: 10.4274/tjod.galenos.2021.90023, 34083750 PMC8191325

[43] Sanguesa J Marquez S Bustamante M Sunyer J Iniguez C Vioque J . Prenatal vitamin D levels influence growth and body composition until 11 years in boys. Nutrients. (2023) 15:2033. doi: 10.3390/nu15092033, 37432159 PMC10181475

[44] Gonzalez TL Willson BE Wang ET Taylor KD Novoa A Swarna A . Sexually dimorphic DNA methylation and gene expression patterns in human first trimester placenta. Biol Sex Differ. (2024) 15:63. doi: 10.1186/s13293-024-00629-9 PMC11328442 39152463

[45] Eriksson JG Kajantie E Osmond C Thornburg K Barker DJP. Boys live dangerously in the womb. Am J Hum Biol. (2010) 22:330–5. doi: 10.1002/ajhb.20995, 19844898 PMC3923652

[46] Olney KC Plaisier SB Phung TN Silasi M Perley L O’Bryan J . Sex differences in early and term placenta are conserved in adult tissues. Biol Sex Differ. (2022) 13:74. doi: 10.1186/s13293-022-00470-y PMC9773522 36550527

[47] Gynecologists ACoOa. ACOG committee opinion no. 495: vitamin D: screening and supplementation during pregnancy. Obstet Gynecol. (2011) 118:197–198. doi: 10.1097/AOG.0b013e318227f06b 21691184

[48] Health Management Branch of Chinese Nutrition Society TS. Expert consensus on evaluation and improvement of vitamin D nutritional status. Chin J Health Manag. (2023) 17:245–252. doi: 10.3760/cma.j.cn115624-20230105-00009

[49] Organization WH Vitamin D Supplementation During Pregnancy – Evidence for Antenatal Care Interventions Geneva, Switzerland: World Health Organization (2023) Available online at: https://www.who.int/tools/elena/interventions/vitamind-supp-pregnancy, (Accessed December 20, 2025).

[50] NHS. Vitamins, supplements and nutrition in pregnancy 2023. (2023) Available online at: https://www.nhs.uk/pregnancy/keeping-well/vitamins-supplements-and-nutrition/ (Accessed December 22, 2025).

[51] Itkonen ST Andersen R Bjork AK Brugard Konde A Eneroth H Erkkola M . Vitamin D status and current policies to achieve adequate vitamin D intake in the Nordic countries. Scand J Public Health. (2021) 49:616–27. doi: 10.1177/1403494819896878, 31916497

