# Supplementary material for: Nonlinear and sex-specific associations of maternal vitamin D in early- and mid-pregnancy with childhood growth trajectories from birth to 6 years of age: a prospective cohort study in China
Source: Front Nutr. 2026 Apr 24;13:1781274. doi: 10.3389/fnut.2026.1781274 (PMC13152784; doi:10.3389/fnut.2026.1781274)
Supplement: Supplementary file 1 [file Table_1.DOCX]

Supplementary Material


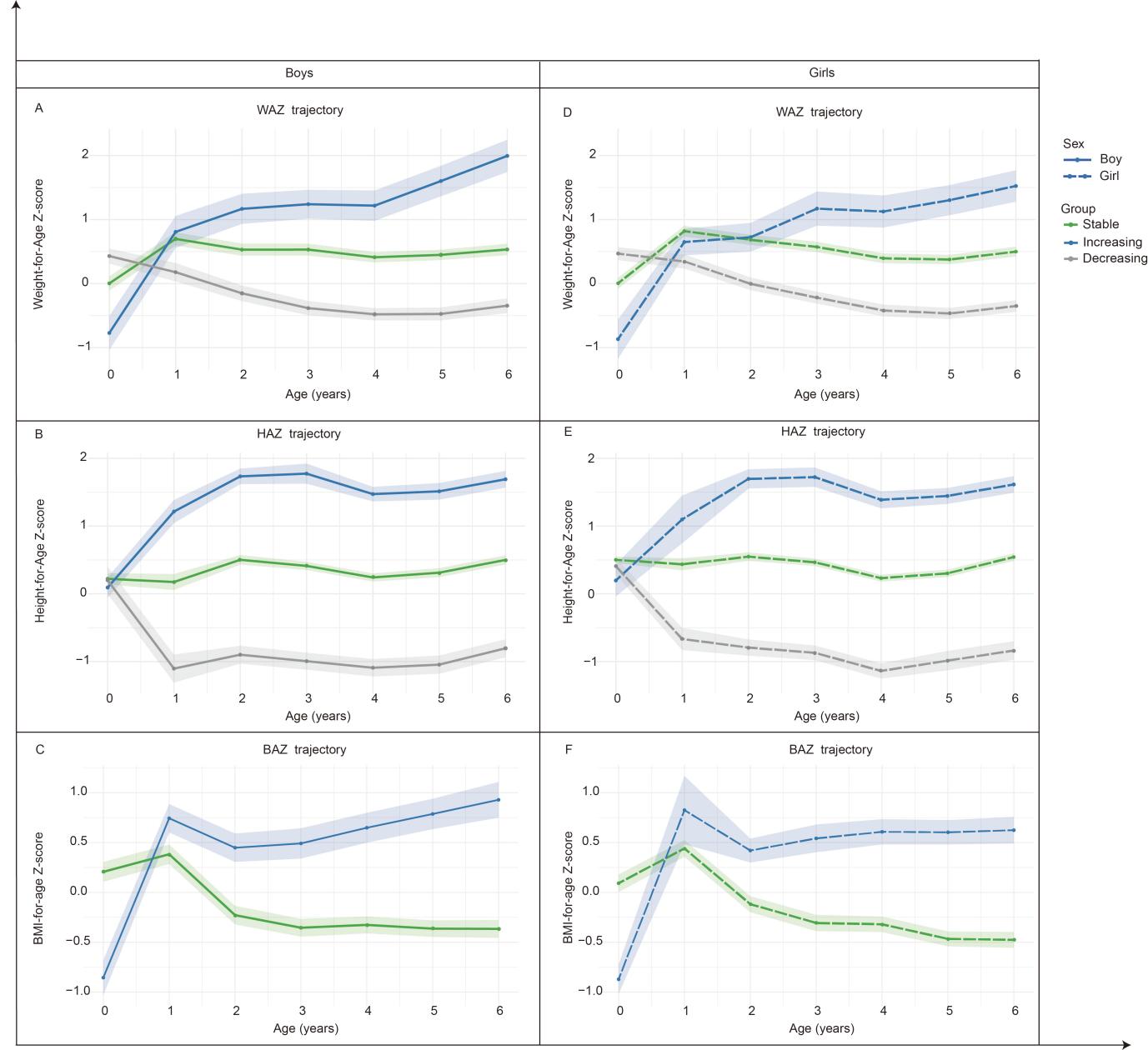


**Supplementary Figure 1.** **Growth trajectories of WAZ, HAZ, and BAZ from birth to 6 years of age (stratified by sex)**

(A–C) Boys: trajectories of weight-for-age z-score (WAZ, A), height-for-age z-score (HAZ, B), and BMI-for-age z-score (BAZ, C). (D–F) Girls: trajectories of WAZ (D), HAZ (E), and BAZ (F).
Shaded areas indicate 95% confidence intervals. Colors denote trajectory patterns: Stable (green), Increasing (blue), and Decreasing (gray, for WAZ and HAZ only).

**
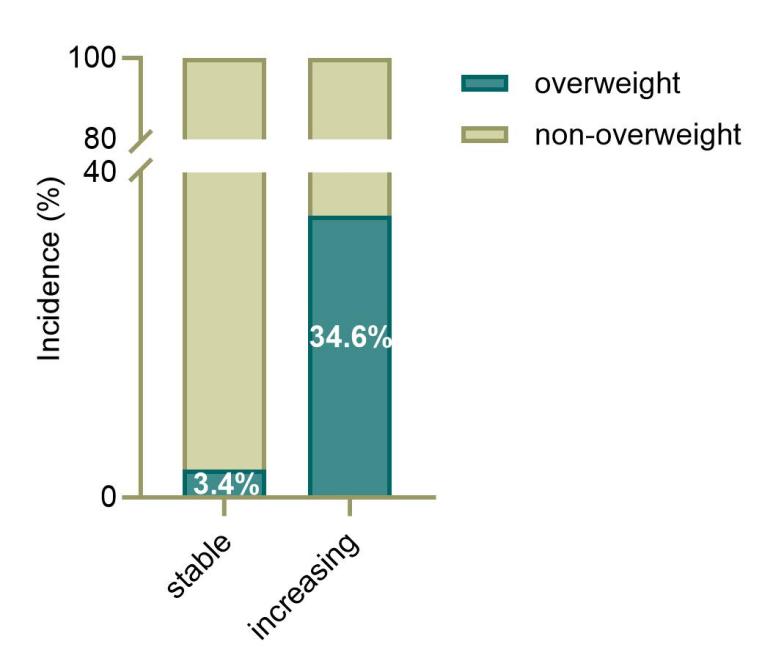
**

**Supplementary Figure 2 Proportions of overweight and non-overweight children at age 6 years by BAZ trajectory group**

**Supplementary Table 1 BIC and 2∆BIC used for model selection and posterior probability for model goodness of fit**

| **Number of groups** | **BIC (N=212)** | **2∆BIC** | **Trajectory size ≥ 5%** | **Average posterior probability in each group** | | | | |
| --- | --- | --- | --- | --- | --- | --- | --- | --- |
|  |  |  |  | **1** | **2** | **3** | **4** | **5** |
| **Weight-for-age z-score** |  |  |  |  |  |  |  |  |
| 1 | 13839.85 |  |  |  |  |  |  |  |
| 2 | 13525.10 | 629.49 | √ | 0.95 | 0.85 |  |  |  |
| 3 | 13451.73 | 776.23 | √ | 0.76 | 0.79 | 0.84 |  |  |
| 4 | 13415.12 | 849.45 | × | 0.79 | 0.76 | 0.78 | 0.83 |  |
| 5 | 13377.87 | 923.96 | × | 0.79 | 0.78 | 0.78 | 0.78 | 0.84 |
| **Length-for-age z-score** |  |  |  |  |  |  |  |  |
| 1 | 13937.77 |  |  |  |  |  |  |  |
| 2 | 13601.78 | 672.00 | √ | 0.86 | 0.85 |  |  |  |
| 3 | 13509.73 | 856.09 | √ | 0.83 | 0.86 | 0.83 |  |  |
| 4 | 13287.81 | 1299.93 | × | 0.85 | 0.87 | 0.96 | 0.85 |  |
| 5 | 13275.79 | 1323.97 | × | 0.88 | 0.84 | 0.79 | 0.97 | 0.81 |
| **BMI-for-age z-score** |  |  |  |  |  |  |  |  |
| 1 | 16378.02 |  |  |  |  |  |  |  |
| 2 | 16148.50 | 459.04 | √ | 0.93 | 0.79 |  |  |  |
| 3 | 16088.87 | 578.31 | × | 0.91 | 0.78 | 0.88 |  |  |
| 4 | 16073.69 | 608.66 | × | 0.73 | 0.74 | 0.82 | 0.89 |  |
| 5 | 16084.11 | 587.83 | × | 0.70 | 0.80 | 0.71 | 0.89 | 0.88 |

BIC = Bayesian information criterion

2∆BIC: between the current model and the null model. A value lower than 2 means no enough evidence against the null model.

Trajectory size was also greater than or equal to 5% of the sample.

Average posterior probability in each group should be ≥ 0.70.

| Supplementary Table 2 Distribution of maternal serum vitamin D concentrations (nmol/L) during early and mid-pregnancy: 33rd and 66th percentiles and corresponding tertile cut-off values   \| **Gestational Period** \| **33rd Percentile (nmol/L)** \| **66th Percentile (nmol/L)** \| **Tertile cut-off values (nmol/L)** \| \| --- \| --- \| --- \| --- \| \| Early pregnancy \| 29.5 \| 42 \| T1 ≤ 29.5; T2 = 29.5–42; T3 > 42 \| \| Mid pregnancy \| 34.7 \| 51.8 \| T1 ≤ 34.7; T2 = 34.7–51.8; T3 > 51.8 \| |
| --- | --- | --- | --- | --- | --- | --- | --- | --- | --- | --- | --- | --- |

T1, T2, and T3 represent the lowest, middle, and highest tertiles of maternal serum vitamin D concentrations, respectively, defined based on the sample distribution for each gestational period.

**Supplementary Table 3** **Baseline characteristics of participants with and without vitamin D (VitD) measurements in early and mid-pregnancy [n (%)]/ (Mean±SD)**

| **Characteristics** | | **Early pregnancy (n=1100)** | | | **Mid pregnancy (n=1100)** | | |
| --- | --- | --- | --- | --- | --- | --- | --- |
|  |  | **With VitD measurement (n=1009)** | **Without VitD measurement (n=91)** | ***P*** | **With VitD measurement(n=588)** | **Without VitD measurement (n=512)** | ***P*** |
| **Maternal** |  |  |  |  |  |  |  |
| 25OHD (nmol/L) | **-** | 37.03±14.27 | - | - | 43.10±20.43 | - | - |
| Maternal age (y) | < 25 | 77(7.63) | 5(5.50) | 0.078 | 38(6.50) | 44(8.60) | 0.362 |
|  | ≥ 25,< 35 | 885(87.71) | 77(84.60) |  | 518(88.10) | 444(86.70) |  |
|  | ≥ 35 | 47(4.66) | 9(9.90) |  | 32(5.40) | 24(4.70) |  |
| Pre-pregnancy BMI (kg/m^2^) | Wasting | 153(15.16) | 6(6.60) | 0.114 | 82(13.90) | 77(15.00) | 0.870 |
|  | Normal | 678(67.20) | 68(74.70) |  | 405(68.90) | 341(66.60) |  |
|  | Overweight | 144(14.27) | 13(14.30) |  | 82(13.90) | 75(14.60) |  |
|  | Obesity | 34(3.37) | 4(4.40) |  | 19(3.20) | 19(3.70) |  |
| Gestational weight gain | Excessive | 213(21.11) | 22(24.20) | 0.214 | 114(19.40) | 121(23.60) | 0.157 |
|  | Suitable | 414(41.03) | 43(47.30) |  | 244(41.50) | 213(41.60) |  |
|  | Too few | 382(37.86) | 26(28.60) |  | 230(39.10) | 178(34.80) |  |
| Educational level (y) | < 9 | 84(8.33) | 12(13.20) | 0.121 | 52(8.84) | 44(8.60) | 0.989 |
|  | ≥ 9, < 16 | 477(47.27) | 47(51.60) |  | 280(47.62) | 244(47.70) |  |
|  | ≥ 16 | 448(44.40) | 32(35.20) |  | 256(43.54) | 224(43.80) |  |
| Per capita household income(CNY) | < 2500 | 143(14.17) | 13(14.30) | 0.950 | 87(14.80) | 69(13.50) | 0.802 |
|  | ≥ 2500,< 5000 | 712(70.56) | 63(69.20) |  | 410(69.70) | 365(71.30) |  |
|  | ≥ 5000 | 154(15.26) | 15(16.50) |  | 91(15.50) | 78(15.20) |  |
| Gravidity | Primigravida | 484(48.00) | 49(53.80) | 0.283 | 280(47.60) | 253(49.40) | 0.552 |
|  | Multigravida | 525(52.00) | 42(46.20) |  | 308(52.40) | 259(50.60) |  |
| Parity | Nulliparous | 700(69.38) | 63(69.20) | 0.977 | 400(68.00) | 363(70.90) | 0.303 |
|  | Multiparous | 309(30.62) | 28(30.80) |  | 188(32.00) | 149(29.10) |  |
| Mode of delivery | Vaginal delivery | 585(57.98) | 49(53.80) | 0.445 | 347(59.00) | 287(56.10) | 0.322 |
|  | Cesarean section | 424(42.02) | 42(46.20) |  | 241(41.00) | 225(43.90) |  |
| VitD supplementation | No | 494 (48.96) | 42(46.20) | 0.608 | 295(50.20) | 241(47.10) | 0.305 |
|  | Yes | 515 (51.04) | 49(53.80) |  | 293(49.80) | 271(52.90) |  |
| Folic acid supplementation | No | 438(43.41) | 33(36.30) | 0.187 | 233(39.60) | 238(46.50) | 0.022* |
|  | Yes | 571(56.59) | 58(63.70) |  | 355(60.40) | 274(53.50) |  |
| Passive smoking in gestation | No | 830(82.30) | 80(87.90) | 0.172 | 478(81.30) | 432(84.40) | 0.177 |
|  | Yes | 179(17.70) | 11(12.10) |  | 110(18.70) | 80(15.60) |  |
| **Child** |  |  |  |  |  |  |  |
| Gestational age (weeks) | - | 38.90±1.41 | 38.63±1.84 | 0.447 | 38.92±1.39 | 38.82±1.51 | 0.235 |
| Birth weight (kg) | - | 3.31±0.44 | 3.26±0.56 | 0.324 | 3.31±0.44 | 3.33±0.45 | 0.875 |
| Infant sex | Boy | 477(47.27) | 45(49.50) | 0.691 | 270(45.90) | 252(49.20) | 0.274 |
|  | Girl | 532(52.73) | 46(50.50) |  | 318(54.10) | 260(50.80) |  |
| Season of delivery | Spring | 298(29.53) | 20(22.00) | 0.014* | 155(26.40) | 163(31.80) | 0.081 |
|  | Summer | 178(17.64) | 10(11.00) |  | 98(16.70) | 90(17.60) |  |
|  | Autumn | 261(25.87) | 37(40.70) |  | 176(29.90) | 122(23.80) |  |
|  | Winter | 272(26.96) | 24(26.40) |  | 159(27.00) | 137(26.80) |  |

* *P* < 0.05.

**Supplementary Table 4 Baseline characteristics of participants according to availability of anthropometric measurements at the 2-year follow-up [n (%)]/ (Mean±SD)**

| **Characteristics** | | **Available at 2 years(n=751)** | **Missing at 2 years (n=349)** | ***P* value** |
| --- | --- | --- | --- | --- |
| **Maternal** |  |  |  |  |
| 25OHD in early pregnancy (nmol/L) | - | 36.8±14.2 | 37.5±14.3 | 0.442 |
| 25OHD in mid pregnancy (nmol/L) | - | 45.3±18.4 | 44.5±19.0 | 0.617 |
| Maternal age (y) | <25 | 50(6.7) | 32(9.2) | 0.194 |
|  | ≥25,<35 | 666(88.7) | 296(84.8) |  |
|  | ≥35 | 35(4.6) | 21(6.0) |  |
| Pre-pregnancy BMI (kg/m^2^) | Wasting | 116(15.4) | 43(12.3) | 0.004 |
|  | Normal | 514(68.4) | 232(66.5) |  |
|  | Overweight | 90(12.0) | 67(19.2) |  |
|  | Obesity | 31(4.1) | 7(2.0) |  |
| Gestational weight gain | Excessive | 159(21.2) | 76(21.8) | 0.252 |
|  | Suitable | 324(43.1) | 133(38.1) |  |
|  | Too few | 268(35.7) | 140(40.1) |  |
| Educational level (y) | <9 | 57(7.6) | 39(11.2) | 0.140 |
|  | ≥9,<16 | 360(47.9) | 164(47.0) |  |
|  | ≥16 | 334(44.5) | 146(41.8) |  |
| Per capita household income(CNY） | <2500 | 104(13.8) | 52 (14.9) | 0.897 |
|  | ≥2500,<5000 | 531(70.7) | 244 (69.9) |  |
|  | ≥5000 | 116(15.4) | 53 (15.2) |  |
| Gravidity | Primigravida | 358(47.7) | 168(48.1) | 0.885 |
|  | Multigravida | 393(52.3) | 181(51.9) |  |
| Parity | Nulliparous | 533(71.0) | 230 (65.9) | 0.104 |
|  | Multiparous | 218(29.0) | 119 (34.1) |  |
| Mode of delivery | Vaginal delivery | 443(59.0) | 191 (54.7) | 0.206 |
|  | Cesarean section | 308(41.0) | 158 (45.3) |  |
| VitD supplementation | No | 360(47.9) | 176 (50.4) | 0.481 |
|  | Yes | 391(52.1) | 173 (49.6) |  |
| Folic acid supplementation | No | 315(41.9) | 156 (44.7) | 0.427 |
|  | Yes | 436(58.1) | 193 (55.3) |  |
| Passive smoking in gestation | No | 627(83.5) | 283(81.1) | 0.327 |
|  | Yes | 124(16.5) | 66(18.9) |  |
| **Child** |  |  |  |  |
| Gestational age (weeks) | - | 38.9±1.4 | 38.8±1.5 | 0.145 |
| Birth weight (kg) | - | 3.3±0.4 | 3.3±0.46 | 0.504 |
| Infant sex | Boy | 353(47.0) | 169 (48.4) | 0.708 |
|  | Girl | 398(53.0) | 180 (51.6) |  |
| Season of delivery | Spring | 210(28.0) | 108 (30.9) | 0.007 |
|  | Summer | 135(18.0) | 53 (15.2) |  |
|  | Autumn | 222(29.6) | 76 (21.8) |  |
|  | Winter | 184(24.5) | 112 (32.1) |  |

Data are presented as n (%) for categorical variables and mean ± SD for continuous variables. Group differences were assessed using chi-square tests or independent t-tests, as appropriate. Participants were classified according to the availability of anthropometric measurements at the 2-year follow-up.

Supplementary Table 5 Sensitivity analysis using complete-case data for associations between maternal vitamin D tertiles during early and mid-pregnancy and offspring growth trajectory groups (WAZ, HAZ, and BAZ) from ages 1–6 years

| **Trajectory Groups** | **Early pregnancy (n=721)** | | | | **Mid pregnancy (n=435)** | | | |
| --- | --- | --- | --- | --- | --- | --- | --- | --- |
|  | **Main analysis** | | **Sensitivity analysis** | | **Main analysis** | | **Sensitivity analysis** | |
|  | Adjusted OR  (95% CI) | *P* value | Adjusted OR  (95% CI) | *P* value | Adjusted OR  (95% CI) | *P* value | Adjusted OR  (95% CI) | *P* value |
| **T 1 vs T 2** |  |  |  |  |  |  |  |  |
| **WAZ** |  |  |  |  |  |  |  |  |
| Stable | Reference |  | Reference |  | Reference |  | Reference |  |
| Increasing | 1.48 (0.84-2.62) | 0.179 | 0.80 (0.44-1.48) | 0.480 | 3.21 (1.41-7.34) | 0.006 | 1.41 (0.57-3.45) | 0.454 |
| Decreasing | 1.00 (0.55-1.83) | 0.507 | 0.75 (0.50-1.12) | 0.165 | 1.17 (0.70-1.95) | 0.551 | 0.94 (0.57-1.56) | 0.818 |
| **HAZ** |  |  |  |  |  |  |  |  |
| Stable | Reference |  | Reference |  | Reference |  | Reference |  |
| Increasing | 1.84 (1.16-2.92) | 0.009 | 1.30 (0.81-2.10) | 0.278 | 1.94 (1.03-3.66) | 0.039 | 1.74 (0.93-3.25) | 0.085 |
| Decreasing | 1.58 (0.88-2.84) | 0.122 | 1.44 (0.81-2.58) | 0.218 | 1.55 (0.72-3.32) | 0.263 | 1.32 (0.62-2.81) | 0.476 |
| **BAZ** |  |  |  |  |  |  |  |  |
| Stable | Reference |  | Reference |  | Reference |  | Reference |  |
| Increasing | 1.63 (1.09-2.43) | 0.016 | 1.48 (1.00-2.20) | 0.051 | 1.29 (0.77-2.15) | 0.336 | 0.83 (0.49-1.41) | 0.491 |
| **T 3 vs T 2** |  |  |  |  |  |  |  |  |
| **WAZ** |  |  |  |  |  |  |  |  |
| Stable | Reference |  | Reference |  | Reference |  | Reference |  |
| Increasing | 1.00 (0.55-1.83) | 0.992 | 1.56 (0.89-2.74) | 0.118 | 1.46 (0.60-3.55) | 0.408 | 2.51 (1.08-5.85) | 0.032 |
| Decreasing | 0.78 (0.52-1.17) | 0.225 | 0.84 (0.56-1.26) | 0.395 | 0.78 (0.47-1.30) | 0.342 | 0.85 (0.51-1.42) | 0.524 |
| **HAZ** |  |  |  |  |  |  |  |  |
| Stable | Reference |  | Reference |  | Reference |  | Reference |  |
| Increasing | 1.23 (0.77-1.99) | 0.389 | 1.99 (1.26-3.15) | 0.003 | 1.53 (0.80-2.93) | 0.203 | 1.63 (0.87-3.08) | 0.130 |
| Decreasing | 1.05 (0.57-1.92) | 0.882 | 1.17 (0.64-2.15) | 0.617 | 1.45 (0.67-3.13) | 0.339 | 1.38 (0.65-2.93) | 0.409 |
| **BAZ** |  |  |  |  |  |  |  |  |
| Stable | Reference |  | Reference |  | Reference |  | Reference |  |
| Increasing | 1.55 (1.04-2.31) | 0.031 | 1.54 (1.04-2.28) | 0.032 | 1.03 (0.61-1.75) | 0.903 | 1.68 (1.01-2.79) | 0.044 |

Odds ratios (ORs) and 95% confidence intervals (CIs) were estimated using multinomial logistic regression models. T1 and T3 were compared with the middle tertile (T2, reference), and the stable trajectory group was used as the reference outcome.Complete-case analysis included participants with no missing data on exposure, outcomes, or covariates. Models were adjusted for the same covariates as in the main analysis.

**Supplementary Table 6 Adjusted odds ratios (95% CI) for offspring elevated BMI-for-age Z-score (BAZ > +1 SD) at ages 1–6 years according to tertiles of maternal vitamin D during early and mid pregnancy**

| **Age (years)** | **Early pregnancy** | |  | **Mid pregnancy** | |  |
| --- | --- | --- | --- | --- | --- | --- |
|  | **Adjusted OR (95% CI)** | ***P* value** | ***P* -FDR** | **Adjusted OR (95% CI)** | ***P* value** | ***P* -FDR** |
| **T1 vs T2** |  |  |  |  |  |  |
| 1 | 1.478 (0.963-2.278) | 0.075 | 0.360 | 1.398 (0.787-2.502) | 0.255 | 0.686 |
| 2 | 0.453 (0.203-0.969) | 0.046 | 0.340 | 0.916 (0.309-2.686) | 0.872 | 0.872 |
| 3 | 1.276 (0.679-2.423) | 0.451 | 0.686 | 1.338 (0.540-3.427) | 0.533 | 0.711 |
| 4 | 0.758 (0.371-1.528) | 0.439 | 0.686 | 3.099 (1.010-10.665) | 0.057 | 0.340 |
| 5 | 0.784 (0.409-1.488) | 0.458 | 0.686 | 1.733 (0.644-4.943) | 0.285 | 0.686 |
| 6 | 0.805 (0.458-1.409) | 0.449 | 0.686 | 1.389 (0.593-3.346) | 0.453 | 0.686 |
| **T3 vs T2** |  |  |  |  |  |  |
| 1 | 1.073 (0.695-1.657) | 0.751 | 0.823 | 1.329 (0.752-2.363) | 0.329 | 0.686 |
| 2 | 0.909 (0.452-1.825) | 0.789 | 0.823 | 1.148 (0.422-3.107) | 0.784 | 0.823 |
| 3 | 1.306 (0.693-2.490) | 0.410 | 0.686 | 1.555 (0.634-3.953) | 0.340 | 0.686 |
| 4 | 1.238 (0.649-2.385) | 0.518 | 0.711 | 4.220 (1.416-14.368) | 0.014 | 0.177 |
| 5 | 1.124 (0.619-2.052) | 0.701 | 0.823 | 2.260 (0.854-6.408) | 0.109 | 0.436 |
| 6 | 1.145 (0.674-1.952) | 0.616 | 0.778 | 2.758 (1.247-6.439) | 0.015 | 0.177 |

Odds ratios (ORs) and 95% confidence intervals (CIs) were estimated using logistic regression models. T1 and T3 were compared with the middle tertile (T2, reference). Models were adjusted for maternal age, pre-pregnancy BMI, gestational weight gain, education, household income, parity, delivery mode, gestational age, infant feeding patterns, and child-related factors at age 1 year (vitamin D supplementation, outdoor time, and sleep duration). P-FDR represents P values adjusted for multiple comparisons using the false discovery rate method.

.

Supplementary Table 7 Overweight prevalence at age 6 years according to BAZ trajectory group

| **BAZ trajectory** | **n** | **Overweight (%)** | **Non-overweight (%)** |
| --- | --- | --- | --- |
| stable | 722 | 25 (3.4) | 697 (96.6) |
| increasing | 378 | 131 (34.6) | 247 (65.4) |

Overweight was defined as BMI-for-age Z-score (BAZ) > +1 SD at age 6 years. Trajectory groups were derived based on longitudinal BAZ measurements from ages 1 to 6 years.

Supplementary Table 8 Sex-stratified adjusted odds ratios (95% CI) for offspring elevated BMI-for-age Z-score (BAZ > +1 SD) at ages 1–6 years according to tertiles of maternal vitamin D during early and mid pregnancy

| **Age (years)** | **Early pregnancy** | |  | **Mid pregnancy** | |  |
| --- | --- | --- | --- | --- | --- | --- |
|  | **Adjusted OR (95% CI)** | ***P* value** | ***P* -FDR** | **Adjusted OR (95% CI)** | ***P* value** | ***P* -FDR** |
| **Boys** |  |  |  |  |  |  |
| **T 1 vs T 2** |  |  |  |  |  |  |
| 1 | 1.494 (0.787-2.864) | 0.222 | 0.675 | 1.664 (0.650-4.389) | 0.292 | 0.675 |
| 2 | 0.869 (0.279-2.650) | 0.804 | 0.919 | 2.189 (0.389-16.523) | 0.397 | 0.763 |
| 3 | 1.283 (0.501-3.351) | 0.604 | 0.906 | 3.188 (0.644-21.193) | 0.182 | 0.671 |
| 4 | 0.452 (0.138-1.348) | 0.165 | 0.671 | 1.330 (0.204-10.176) | 0.768 | 0.917 |
| 5 | 0.757 (0.304-1.843) | 0.542 | 0.882 | 1.306 (0.290-6.257) | 0.728 | 0.917 |
| 6 | 0.745 (0.341-1.606) | 0.454 | 0.838 | 1.319 (0.412-4.362) | 0.641 | 0.906 |
| **T 3 vs T 2** |  |  |  |  |  |  |
| 1 | 1.033 (0.543-1.969) | 0.921 | 0.977 | 3.516 (1.284-10.408) | 0.018 | 0.303 |
| 2 | 0.911 (0.309-2.667) | 0.863 | 0.942 | 10.648 (1.178-159.716) | 0.054 | 0.430 |
| 3 | 1.182 (0.450-3.161) | 0.734 | 0.917 | 4.600 (0.801-36.235) | 0.109 | 0.580 |
| 4 | 0.739 (0.272-1.996) | 0.549 | 0.882 | 2.766 (0.322-30.918) | 0.372 | 0.744 |
| 5 | 0.853 (0.346-2.084) | 0.726 | 0.917 | 2.364 (0.548-11.981) | 0.267 | 0.675 |
| 6 | 1.030 (0.480-2.218) | 0.940 | 0.977 | 1.883 (0.592-6.514) | 0.295 | 0.675 |
| **Girls** |  |  |  |  |  |  |
| **T 1 vs T 2** |  |  |  |  |  |  |
| 1 | 1.691 (0.911-3.180) | 0.099 | 0.580 | 1.197 (0.527-2.745) | 0.667 | 0.915 |
| 2 | 0.308 (0.087-0.976) | 0.053 | 0.430 | 0.649 (0.103-3.764) | 0.630 | 0.906 |
| 3 | 1.713 (0.650-4.765) | 0.284 | 0.675 | 0.630 (0.151-2.583) | 0.517 | 0.882 |
| 4 | 1.354 (0.485-3.907) | 0.565 | 0.882 | 3.930 (0.553-41.529) | 0.202 | 0.675 |
| 5 | 1.101 (0.391-3.139) | 0.855 | 0.942 | 9.342 (0.936-179.597) | 0.089 | 0.580 |
| 6 | 0.976 (0.400-2.365) | 0.956 | 0.977 | 2.919 (0.492-23.037) | 0.265 | 0.675 |
| **T 3 vs T 2** |  |  |  |  |  |  |
| 1 | 0.997 (0.529-1.881) | 0.993 | 0.993 | 0.795 (0.357-1.753) | 0.569 | 0.882 |
| 2 | 1.167 (0.387-3.516) | 0.782 | 0.917 | 0.501 (0.101-2.150) | 0.364 | 0.744 |
| 3 | 1.973 (0.757-5.482) | 0.174 | 0.671 | 1.197 (0.331-4.483) | 0.783 | 0.917 |
| 4 | 2.109 (0.787-5.984) | 0.146 | 0.671 | 10.687 (1.877-111.578) | 0.019 | 0.303 |
| 5 | 1.568 (0.594-4.314) | 0.369 | 0.744 | 16.670 (1.823-351.610) | 0.033 | 0.390 |
| 6 | 1.628 (0.708-3.824) | 0.255 | 0.675 | 14.719 (2.963-114.512) | 0.003 | 0.155 |

Odds ratios (ORs) for T1 and T3 are shown relative to the middle tertile (T2, reference). All models were adjusted for maternal age, pre-pregnancy body mass index (BMI), gestational weight gain, maternal educational level, per capita household income, parity, mode of delivery, gestational age at birth, infant feeding patterns, and child-related covariates at 1 year of age, including vitamin D supplementation, daily outdoor time, and daily sleep duration. P-FDR represents P values adjusted for multiple comparisons using the false discovery rate method.

**Supplementary Table 9 Sensitivity analysis using complete-case data for associations between maternal vitamin D tertiles during early and mid-pregnancy and offspring overweight/obesity risk at ages 1–6 years**

| **Age (years)** | **Early pregnancy(n=721)** | | **Mid pregnancy(n=435)** | |
| --- | --- | --- | --- | --- |
|  | **Adjusted OR (95% CI)** | ***P* value** | **Adjusted OR (95% CI)** | ***P* value** |
| **T1 vs T2** |  |  |  |  |
| 1 | 1.16 (0.77-1.76) | 0.486 | 1.12 (0.64-1.94) | 0.692 |
| 2 | 1.15 (0.69-1.91) | 0.601 | 1.31 (0.68-2.54) | 0.423 |
| 3 | 0.89 (0.53-1.47) | 0.637 | 1.51 (0.78-2.99) | 0.228 |
| 4 | 0.83 (0.47-1.44) | 0.508 | 1.96 (0.88-4.51) | 0.103 |
| 5 | 0.63 (0.37-1.06) | 0.084 | 2.27 (1.03-5.22) | 0.046 |
| 6 | 0.80 (0.49-1.30) | 0.375 | 1.25 (0.64-2.46) | 0.523 |
| **T3 vs T2** |  |  |  |  |
| 1 | 1.20 (0.80-1.81) | 0.372 | 1.45 (0.84-2.50) | 0.182 |
| 2 | 1.17 (0.71-1.96) | 0.539 | 1.06 (0.54-2.08) | 0.857 |
| 3 | 1.78 (1.11-2.88) | 0.018 | 1.30 (0.66-2.59) | 0.445 |
| 4 | 1.52 (0.92-2.55) | 0.108 | 2.19 (1.00-4.96) | 0.053 |
| 5 | 1.14 (0.70-1.84) | 0.600 | 3.01 (1.38-7.01) | 0.007 |
| 6 | 1.21 (0.76-1.92) | 0.426 | 2.32 (1.22-4.50) | 0.011 |

Odds ratios (ORs) and 95% confidence intervals (CIs) were estimated using logistic regression models. T1 and T3 were compared with the middle tertile (T2, reference). Complete-case analysis included participants with no missing data on exposure, outcomes, or covariates. Models were adjusted for the same covariates as in the main analysis.

Supplementary Table 10 Offspring overweight/obesity risk at ages 1–6 years by maternal vitamin D tertiles, complete-case analysis, stratified by sex

| **Age (years)** | **Early pregnancy** | |  | **Mid pregnancy** | |  |
| --- | --- | --- | --- | --- | --- | --- |
|  | **Adjusted OR (95% CI)** | ***P* value** | **N** | **Adjusted OR (95% CI)** | ***P* value** | **N** |
| **Boys** |  |  |  |  |  |  |
| **T 1 vs T 2** |  |  |  |  |  |  |
| 1 | 0.86 (0.46–1.61) | 0.632 | 342 | 0.87 (0.35–2.15) | 0.758 | 192 |
| 2 | 0.86 (0.41–1.80) | 0.690 | 342 | 0.68 (0.23–2.00) | 0.488 | 192 |
| 3 | 1.08 (0.49–2.38) | 0.843 | 342 | 1.45 (0.44–5.02) | 0.546 | 192 |
| 4 | 0.73 (0.33–1.60) | 0.439 | 342 | 1.20 (0.31–4.86) | 0.793 | 192 |
| 5 | 0.60 (0.28–1.24) | 0.174 | 342 | 2.26 (0.66–8.63) | 0.208 | 192 |
| 6 | 0.70 (0.35–1.36) | 0.293 | 342 | 0.82 (0.29–2.26) | 0.698 | 192 |
| **T 3 vs T 2** |  |  |  |  |  |  |
| 1 | 0.97 (0.52–1.80) | 0.919 | 342 | 1.92 (0.75–5.14) | 0.182 | 192 |
| 2 | 0.77 (0.36–1.64) | 0.502 | 342 | 1.69 (0.53–5.73) | 0.379 | 192 |
| 3 | 1.88 (0.89–4.07) | 0.103 | 342 | 1.87 (0.50–7.69) | 0.364 | 192 |
| 4 | 1.00 (0.47–2.13) | 0.99 | 342 | 1.23 (0.31–5.29) | 0.774 | 192 |
| 5 | 0.99 (0.49–1.99) | 0.967 | 342 | 2.49 (0.69–10.10) | 0.176 | 192 |
| 6 | 0.90 (0.46–1.76) | 0.753 | 342 | 1.66 (0.59–4.89) | 0.342 | 192 |
| **Girls** |  |  |  |  |  |  |
| **T 1 vs T 2** |  |  |  |  |  |  |
| 1 | 1.27 (0.71–2.28) | 0.414 | 379 | 0.85 (0.38–1.90) | 0.692 | 243 |
| 2 | 1.29 (0.61–2.77) | 0.512 | 379 | 1.43 (0.56–3.72) | 0.451 | 243 |
| 3 | 0.61 (0.30–1.22) | 0.162 | 379 | 0.86 (0.34–2.17) | 0.747 | 243 |
| 4 | 0.73 (0.32–1.67) | 0.453 | 379 | 1.78 (0.55–6.09) | 0.342 | 243 |
| 5 | 0.55 (0.24–1.23) | 0.149 | 379 | 1.59 (0.50–5.56) | 0.444 | 243 |
| 6 | 0.93 (0.43–2.00) | 0.846 | 379 | 0.86 (0.29–2.56) | 0.788 | 243 |
| **T 3 vs T 2** |  |  |  |  |  |  |
| 1 | 1.20 (0.67–2.67) | 0.342 | 379 | 0.98 (0.46–2.09) | 0.965 | 243 |
| 2 | 1.58 (0.74–3.45) | 0.237 | 379 | 0.65 (0.24–1.70) | 0.382 | 243 |
| 3 | 1.37 (0.71–2.67) | 0.342 | 379 | 0.93 (0.38–2.26) | 0.864 | 243 |
| 4 | 2.06 (0.98–4.49) | 0.061 | 379 | 3.17 (1.09–10.35) | 0.042 | 243 |
| 5 | 1.07 (0.50–2.29) | 0.856 | 379 | 3.87 (1.26–13.62) | 0.024 | 243 |
| 6 | 1.78 (0.87–3.72) | 0.117 | 379 | 3.49 (1.33–9.89) | 0.014 | 243 |

ORs (95% CIs) were estimated by logistic regression, comparing T1 and T3 to T2 (reference). Complete-case analysis included participants with no missing data. Models were adjusted for the same covariates as in the main analysis. Overweight/obesity was defined as BAZ > +1 SD. Overweight/obesity was defined as BAZ > +1 SD.
